# Insights on the Extraction Performance of Alkanediols and Glycerol: Using *Juglans regia* L. Leaves as a Source of Bioactive Compounds

**DOI:** 10.3390/molecules25112497

**Published:** 2020-05-27

**Authors:** Vanessa Vieira, Ricardo C. Calhelha, Lillian Barros, João A. P. Coutinho, Isabel C. F. R. Ferreira, Olga Ferreira

**Affiliations:** 1 CICECO—Aveiro Institute of Materials, Complexo de Laboratórios Tecnológicos, Aveiro University, Campus Universitário de Santiago, 3810-193 Aveiro, Portugal; vanessa.vieira@ua.pt (V.V.); jcoutinho@ua.pt (J.A.P.C.); 2 Laboratory of Separation and Reaction Engineering—Laboratory of Catalysis and Materials (LSRE-LCM), Polytechnic Institute of Bragança, Campus de Santa Apolónia, 5300-253 Bragança, Portugal; 3 Centro de Investigação de Montanha (CIMO), Instituto Politécnico de Bragança, Campus de Santa Apolónia, 5300-253 Bragança, Portugal; calhelha@ipb.pt (R.C.C.); iferreira@ipb.pt (I.C.F.R.F.)

**Keywords:** alkanediols, glycerol, phenolic compounds, *Juglans regia* L., cytotoxicity, anti-inflammatory activity, solvents

## Abstract

Glycerol and alkanediols are being studied as alternative solvents to extract phytochemicals from plant material, often as hydrogen bond donors in deep eutectic solvents (DESs). Many of those alcohols are liquid at room temperature, yet studies of their use as extraction solvents are scarce. In this work, glycerol and a series of alkanediols (1,2-ethanediol, 1,2-propanediol, 1,3-propanediol, 1,3-butanediol, 1,2-pentanediol, 1,5-pentanediol, and 1,2-hexanediol) were studied for the extraction of phenolic compounds from *Juglans regia* L. leaves, a rich source of this class of bioactive compounds. The extraction yield was quantified, and the bioactivity of both extracts and pure solvents was evaluated by measuring the anti-inflammatory and cytotoxic activities. The solvents showing the best combined results were 1,2 and 1,3-propanediol, as their extracts presented a high amount of phenolic compounds, close to the results of ethanol, and similar cytotoxicity against cervical carcinoma cells, with no impact on non-tumor porcine liver cells in the studied concentration range. On the other hand, none of the extracts (and solvents) presented anti-inflammatory activity. Overall, the results obtained in this work contribute to the study of alternative solvents that could potentially be used also as formulation media, highlighting the importance of walnut leaves as a source of bioactive compounds.

## 1. Introduction

Plants are important sources of bioactive phytochemicals, with a wide range of applications in the food, pharmaceutical, and cosmetic sectors as natural ingredients, e.g., replacing synthetic additives or improving the quality of a final product [1]. In particular, the leaves of *Juglans regia* L. (walnut tree) have been reported as a rich source of phenolic compounds, especially hydroxycinnamic acids and flavonols [2,3], with potential application in cosmetics or pharmaceutics [4], and stand out for their bioactive properties (antioxidant, anti-inflammatory, antidiabetic, anti-proliferative, and antibacterial, among others) [5,6].

Plant extracts are frequently obtained by extraction processes using conventional volatile organic solvents. However, alternative solvents that are more efficient, sustainable, and safer are being proposed in the literature, and those include deep eutectic solvents (DES) [7,8]. While the definition of a deep eutectic solvent is still under debate [9], a considerable number of those solvents include components that are liquid at room temperature, such as glycerol, ethylene glycol, propanediols (1,2 and 1,3 isomers), butanediols (1,2, 1,3 and 1,4 isomers), and triethylene glycol, as recently reviewed [10,11,12,13,14,15]. Nevertheless, their application as pure solvents remains scarce. In fact, the use of alternative alcohols as solvents to extract phytochemicals from plants is limited to a few studies using ethylene glycol, propanediols, and glycerol. As can be seen in Table 1, the solvents most commonly applied, often mixed with water, were 1,2-propanediol [16,17,18,19,20,21,22,23,24,25] and glycerol [16,25,26,27,28,29,30], and less frequently ethylene glycol [31].

In the case of systems containing glycerol, higher total phenolic contents were obtained, using small amounts of glycerol in water, from *Olea europaea* leaves (9.3%, *w*/*v*) [28], *Hypericum perforatum* dried aerial parts (10%, *w*/*v*) [26], and *Oryza sativa* bran (20%, *v*/*v*) [30]. On the other hand, an increasing concentration of glycerol (10–90%, *w*/*v*) leads to extracts of two *Artemisia* species with higher total phenolic content [27]. Philippi et al. [29] compared the extraction yields of aqueous solutions of glycerol or ethanol, obtaining similar total phenolic contents in the extracts of *Solanum melongena*. Regarding the use of propylene glycol, in some cases, higher extraction yields were obtained than by using water and/or ethanol. That was the case of the extracts obtained from *Stevia rebaudiana* leaves [22], *Terminalia chebula* fruits [23], *Malus sylvestris* wild fruit [18], and *Pandanus amaryllifolius* leaves [20]. On the other hand, the aqueous propylene glycol extract of *Terminalia chebula* fruits contained lower amounts of gallic and ellagic acids compared to the hydroethanolic extract [24]. Finally, using aqueous solutions of ethylene glycol, a higher total phenolic content was obtained from *Morus alba* L. when compared to acetone + (water or methanol) mixtures [31].

Besides the aqueous solutions of alcohols, other combinations were also studied. Binary mixtures of ethanol + propylene glycol or glycerol were more effective than ethanol for the extraction of rosmarinic acid, carvacrol, oleanolic acid, and ursolic acid from three *Origanun* species by heat-assisted extractions (HAEs) [16], while higher amounts of isorhamnetin-3-*O*-rutinoside were extracted from *Calendula officinalis* L. flowers using an ethanol + propylene glycol + water equimolar mixture than using pure solvents or binary mixtures [17].

In this work, aqueous solutions of glycerol and alkanediols (1,2-ethanediol, 1,2-propanediol, 1,3-propanediol, 1,3-butanediol, 1,2-pentanediol, 1,5-pentanediol, and 1,2-hexanediol) were studied to extract phenolic compounds from walnut leaves. These compounds were chosen not only to evaluate the effect of increasing the alkyl chain of the diols on the extraction yield, but also considering their potential application in different areas such as pharmaceuticals or cosmetics. Some of these compounds and their functions are included in a list of cosmetic ingredients (other than perfume and aromatic raw materials) [32]: ethanol (solvent), 1,2-ethanediol (solvent/viscosity controlling/humectant), 1,2-propanediol (humectant/solvent/skin conditioning/viscosity controlling), 1,3-butanediol (humectant/solvent), and glycerol (denaturant/humectant/solvent). A recent patent published by Lavaud et al. [25] about the use of alternative solvents for food and cosmetic applications also includes 1,2-ethanediol, 1,2-propanediol, 1,3-propanediol, 1,2-pentanediol, and glycerol, highlighting these ingredients as “bio-sourced natural compounds” and the possibility of obtaining them from renewable sources. Finally, 1,5-pentanediol was also studied as a preserving and humectant ingredient for dermatological care products [33,34,35], and 1,2-hexanediol was proposed as a potential component for the preparation of topical formulations aiming to retard the percutaneous absorption of active pharmaceutical ingredients [36]. On an industrial scale, the use of alkanediols and glycerol could be also safer as they have lower vapor pressures compared to conventional organic solvents [37,38,39]. In addition, if the solvent could be part of the final formulation, the number of unit operations of the global process would be reduced by eliminating the need to separate the extracted compounds from the solvent [15,37]. Finally, to support the development of new applications, besides determining the extraction yield of the main phenolic compounds, the anti-inflammatory and cytotoxic activities of both extracts and pure solvents were also evaluated.

## 2. Results and Discussion

### 2.1. Extraction Yields

Aqueous solutions of glycerol and several diols (1,2-ethanediol, 1,2-propanediol, 1,3-propanediol, 1,3-butanediol, 1,2-pentanediol, 1,5-pentanediol, and 1,2-hexanediol) containing 20% water (*w*/*w*) were used to extract phenolic compounds from *J. regia* L. leaves. Water and ethanol + water (80:20, *w*/*w*) solvents were also studied for comparison purposes. To carry out this study, aqueous solutions of the solvents referred to were preferred as they have shown to improve the extraction yields by reducing the solvent viscosity, enhancing the mass-transfer from the plant to the solvent media and reducing energy consumption [40]. The optimum amount of water depends on several factors such as the plant matrix or the target molecules to be extracted, as summarized in Table 1. In this work, this amount was selected taking into account a previous study aimed at solvent screening for the extraction of phenolics from the same plant material [41].

To evaluate the extraction efficiency, the amount of the four main phenolic compounds (3-*O*-caffeoylquinic acid, *trans* 3-*p*-coumaroylquinic acid, quercetin-3-*O*-glucoside and quercetin-*O*-pentoside) was quantified by high-performance liquid chromatography with diode-array detection (HPLC-DAD).

The extraction yields of the main phenolic compounds is presented in Table 2. As can be seen, hydroalcoholic solvents (except 1,2-hexanediol) exhibited higher extraction yields than water. This group of diols performed similarly to ethanol (27.8 ± 0.1 mg/g dry plant), the phenolic content ranging from 23.9 ± 0.3 mg/g dry plant (1,3-butanediol) to 30.5 ± 0.2 mg/g dry plant (1,2-ethanediol). The 1,2-ethanediol extract was the only one exceeding the extraction yields obtained by the reference solvent (ethanol). Propanediol extracts (1,2 and 1,3 isomers) have equivalent extraction yields. In contrast, with the more polar glycerol (1,2,3-propanetriol), only a total content of 18.3 ± 0.4 mg/g dry plant was obtained. Regarding the general phenolic profile of pentanediol extracts, the 1,2 isomer extracted the highest amount of quercetin derivatives (24.5 mg/g dry plant), considering all solvents. In contrast, the 1,5-pentanediol liquid extract was richer in phenolic acids (5.69 mg/g dry plant) than the 1,2 isomer (1.60 mg/g dry plant). Finally, 1,2-hexanediol was the poorest solvent (5.73 ± 0.09 mg/g dry plant), with lower extraction yields than water (14.1 ± 0.3 mg/g dry plant).

The individual amounts of each phenolic compound obtained in this work, using conventional ethanol/water (80:20, *w*/*w*) mixtures, are generally in good agreement with the ones reported in the literature, as recently reviewed [42]. In previous studies, similar amounts of 3-*O*-caffeoylquinic acid were found using water as solvent, and also of quercetin 3-*O*-glucoside and quercetin *O*-pentoside using water and ethanol [43], or even eutectic mixtures based on choline chloride and carboxylic acids [41]. On the other hand, the values found in this work were higher than the ones presented by Zhao et al. [44], Amaral et al. [45], and Santos et al. [3], while Pereira et al. [46] reported higher amounts of 3-*O*-caffeoylquinic acid (12.06–14.82 mg/g dw), 3-*p*-coumaroylquinic acid (4.69–5.99 mg/g dw), and quercetin 3-*O*-galactoside (15.72–21.68 mg/g dw) in the aqueous (decoction) extract of different cultivars of walnut leaves. These differences arise from the combination of several factors, namely: (i) the type of solvent; (ii) extraction conditions; (iii) the plant (e.g., geographical location, time of collection, type of cultivar, etc.) [47,48]. In this work, the extraction conditions and the lot of the plant were the same, to allow the evaluation of the effect of changing the solvent, as discussed in the previous paragraph.

### 2.2. Cytotoxic and Anti-Inflammatory Activities

To assist the identification of potential applications for these extracts, cytotoxicity assays were applied not only to the extracts, but also to the solvents. The cytotoxicity was evaluated using a human tumor cell line (HeLa) and the non-tumor porcine live primary cell culture (PLP2). The HeLa cell line represents a suitable in vitro model to study cytotoxicity that has already been applied to new solvents [49] such as DES [50,51] and ionic liquids prepared from biomaterials [52]. On the other hand, PLP2 cells can be used to evaluate one specific type of cytotoxicity-hepatotoxicity [53]. Therefore, the potential toxicity occurring during/after metabolization is here represented by the porcine liver primary cells [54]. The results of the cytotoxicity assays are summarized in Table 3.

It is important to mention that none of the extracts were as active as the positive control (GI_50_ ellipticine = 1.0 ± 0.1 and 2.3 ± 0.2 µg/mL for HeLa and PLP2, respectively). As can be seen, the extracts obtained using standard solvents (water and ethanol) have no toxicological impact in PLP2 cells, allowing normal cell proliferation under concentrations up to 500 µg/mL. The same was observed for the aqueous extract applied to the HeLa cell culture; however, for the ethanolic extract, a GI_50_ of 245 ± 14 µg/mL was obtained. Regarding the extracts showing cytotoxic potential only against the tumor cell line but not the non-tumor one (1,2- and 1,3-propanediol, and 1,3-butanediol), 1,3-propanediol was the most effective solvent (GI_50_ = 216 ± 10 µg/mL and > 500 µg/mL, respectively, for the HeLa and PLP2 cell cultures). Interestingly, the extracts obtained using 1,2-ethanediol and glycerol showed toxicity against the selected control cell culture (PLP2), with GI_50_ values of 142 ± 5 µg/mL and 143 ± 5 µg/mL, respectively. Nevertheless, the pure solvents (without extracts) were absent of cytotoxicity up to concentrations of 4%. Besides the cytotoxicity of 1,2-ethanediol and glycerol extracts against the PLP2 cells, the GI_50_ values for the HeLa cells were lower (GI_50_ = 97 ± 10 µg/mL and 88 ± 4 µg/mL, respectively); thus, the extracts were more active against the cervical human tumor cell line. Finally, the most cytotoxic extract was obtained with 1,2-hexanediol, the concentrations of extract being significantly lower than those obtained with the remaining solvents (GI_50, HeLa_ = 37 ± 2 µg/mL). The same was observed for the pure solvent, constituting a strong cytotoxic agent even when highly diluted (GI_50, PLP2_ = 0.285 ± 0.005% and GI_50, HeLa_ = 0.36 ± 0.02%). This was also observed for both pentanediol isomers, which presented activity for concentrations lower than 1%. The 1,5-pentanediol extract showed significantly higher GI_50_ values against the HeLa cell line (GI_50_ = 212 ± 4 µg/mL) than glycerol and 1,2-ethanediol extracts, but the effective GI_50_ for the PLP2 cells was lower (141 ± 3 µg/mL), which is not desirable as ideally, GI_50, PLP2_ > GI_50, HeLa_.

To complete this discussion, it is important to clarify the contribution of the solvent to the final toxicity of the extract. For that reason, the toxicity of the solvent was evaluated in a concentration range that includes the concentration of the solvent in the liquid extracts studied in the bioactivity studies. When both extracts and solvents presented cytotoxicity (1,2-pentanediol, 1,5-pentanediol, and 1,2-hexanediol), it was found that the concentrations providing cytotoxicity for the pure solvents are similar to those presented by the extracts (HeLa = 0.41%, 0.57%, and 0.10%; PLP2 = 0.63%, 0.38%, and 0.13%, respectively). Thus, the toxicity found is probably due to the presence of the solvent. On the other hand, for the remaining extracts, the bioactivity could be attributed to the bioactive substances in the extract since the solvents do not show toxicity against the studied cell lines up to a 4% concentration, which is higher than the amount of solvent present in the analyzed liquid extracts.

The cytotoxicity of walnut leaf extracts was previously studied by Santos et al. [3] for several cell lines, including HeLa and PLP2 cell cultures. The authors reported the absence of toxicity of extracts obtained by decoction (GI_50_ > 400 µg/mL), a specific type of aqueous extract, which is in good agreement with our results. In contrast, the methanolic extract of the same plant material, as well as those obtained from green and yellow leaves using ethanol + water [42], inhibited the proliferation of HeLa but not PLP2 cells, which also agrees with the results obtained in the present study using conventional solvents.

The evaluation of the cytotoxicity of the liquid extracts obtained with alkanediols and glycerol has seldom been carried out (Table 1). A comparative study of the cytotoxic potential of *Stevia rebaudiana* leaf extracts and the selected solvents (water, ethanol, and aqueous propylene glycol) was performed by Gaweł-Bȩben et al. [22] using skin fibroblast cells as a model to evaluate the extracts’ potential as food and cosmetic ingredients. Generally, the extracts show higher cytotoxic potential than the solvents for the same solvent concentration. Furthermore, the aqueous ethanolic and propylene glycol extracts (between 2% and 5%) promoted a significant decrease in the skin fibroblasts’ viability. Thus, the authors concluded that the potential of those extracts for cosmetic or food formulations are dependent on further studies to establish an appropriate dose, adapted to the desired product.

Finally, in a previous study, the extracts of green leaves of *J. regia* showed anti-inflammatory activity [42]; thus, this assay was also applied here. However, neither the liquid extracts nor solvents showed anti-inflammatory potential at the studied concentration range (EC_50_ > 500 µg/mL or 4% solvent). To the best of our knowledge, this is the first time that this cell-based assay was applied to the selected solvents and extracts.

## 3. Materials and Methods

### 3.1. Standards and Reagents

HPLC-grade acetonitrile, 1,2-ethanediol (99.94%), and glycerol (99.88%) were obtained from Fisher Scientific, Waltham, MA, USA. The standards 5-*O*-caffeoylquinic acid, *p*-coumaric acid, and quercetin 3-*O*-glucoside and the solvent 1,3-butanediol (99.5%) were purchased from Sigma-Aldrich. 1,5-pentanediol (98%) was obtained from ACROS Organics, Morris Plains, NJ, USA. 1,3-propanediol (≥ 99.8%) was supplied by DuPont & Lyle BioProducts, Loudon, TN, USA. 1,2-pentanediol (>98%) was purchased from TCI, Tokyo, Japan, while 1,2-hexanediol (97%) was from Alfa AESAR, Kandel, Germany. 1,2-propanediol (99.5%) was purchased from Panreac, Barcelona, Spain. All the other chemicals were of analytical grade and purchased from common sources.

### 3.2. Plant Material

The *Juglans regia* L. (walnut) dried leaves were purchased from Soria Natural, S.A., Soria, Spain. According to the distributor, the leaves were collected in Soria (Spain) during June 2014 and naturally dried in a room with controlled humidity. Before the extractions, the samples were milled (60 to 20 mesh) and stored in a desiccator protected from light for subsequent assays.

### 3.3. Extraction Methodology

Preparation of solvents: firstly, the amount of water in the organic solvents used to prepare the different solutions was measured using a Metrohm 831 Karl Fisher coulometer, Metrohm, Herisau, Switzerland; (data not shown). For the preparation of the solvents, each component was accurately weighed (±10^−4^ g). Aqueous solutions of different alcohols (ethanol, glycerol, 1,2-ethanediol, 1,2-propanediol, 1,3-propanediol, 1,3-butanediol, 1,2-pentanediol, 1,5-pentanediol, and 1,2-hexanediol) were prepared containing 20% (in weight) of water.

Extraction technique: the heat-assisted extractions (HAE) were performed using heating equipment with magnetic stirring (Carousel 12 Plus Reaction Station™, Radleys Tech, Essex, UK). The powdered samples (0.3 g) were extracted with 10 mL of each solvent for 120 min at 50 °C and 600 rpm. These conditions were set for solvent screening, taking into consideration previous results using, as solvents, mixtures of water and ethanol [43] and aqueous mixtures of choline chloride and carboxylic acids [41]. After extraction, the mixtures were filtered through a Whatman nº 4 paper type (Prat Dumas, Couze-St-Front, France) for further analysis.

### 3.4. Chromatographic Analysis of the Main Phenolic Compounds

Before chromatographic analysis, the samples of the solutions containing the extracts were diluted with water and filtered through 0.2 µm disposable liquid chromatography (LC) filter disks (30 mm, regenerated cellulose; Whatman, Maidstone, UK). The samples were analyzed using a Shimadzu 20A series UFLC (Ultra-Fast Liquid Chromatograph, Shimadzu Corporation, Kyoto, Japan) with a quaternary pump and a photodiode array detector (PDA) coupled to an LC solution software data-processing station. A Waters Spherisorb S3 ODS-2 C_18_, (3 μm, 4.6 mm × 150 mm; Waters Associates, PA, USA) column was used, operating at 35 °C. The chromatographic method was previously described by the authors [43]. A diode array detector (DAD) was used at 280 and 370 nm wavelengths. The target phenolic compounds were identified according to their UV spectra and retention time [55]. For the quantitative analysis, a baseline to valley integration with baseline projection mode was used to calculate peak areas, and external standards were used for quantification. The results were expressed in mg per g of dry plant (mg/g dry plant).

### 3.5. Cytotoxicity

*J. regia* leaf extracts (30 mg/mL) and solvents were studied regarding their inhibitory growth activity of the HeLa (cervical carcinoma) cell line and the non-tumor PLP2 cells (porcine liver primary cells) by using a sulforhodamine B assay. The extracts were diluted to obtain a stock solution of 10 mg/mL, and the concentration of the working solutions ranged from 500 to 7.8 µg/mL, while the concentration of the solvents ranged from 4% to 0.0156%. Ellipticine was used as positive control. The experimental protocol related to the cell cultures was previously described by Barros et al. [55] and Abreu et al. [53]. The concentration needed to reach 50% of the growth inhibition effect (GI_50_) was determined.

### 3.6. Anti-Inflammatory Activity

The walnut leaf extracts (30 mg/mL) and the pure solvents were tested with a lipopolysaccharide-induced nitric oxide (NO) production assay according to Corrêa et al. [56], using a mouse macrophage-like cell line (RAW264.7). The extracts were diluted to obtain a stock solution of 10 mg/mL, and the concentration of the working solutions ranged from 500 to 7.8 µg/mL. Again, the concentration of the solvents ranged from 4% to 0.0156%. Then, the anti-inflammatory activity was assessed by measuring the nitrite concentration in the cell culture medium, using the Griess Reagent System kit. Dexamethasone was used as a positive control. The concentration providing 50% of the inhibition of NO production (EC_50_) was determined.

### 3.7. Statistical Analysis

In the bioactivity assays, duplicates of each extract were assayed, and three repetitions of each methodology were performed, with the results expressed as mean values and standard deviations (SD). One-way ANOVA followed by Tukey’s HSD test (*p* = 0.05) were used to analyze the results. Furthermore, significant differences between two samples were established by applying a student’s *t*-test, with *p* = 0.05 using the IBM SPSS Statistics for Windows, Version 23.0. (IBM Corp., Armonk, New York, NY, USA).

## 4. Conclusions

Aqueous solutions of several alkanediols were able to extract similar amounts of phenolic compounds to ethanol, a conventional volatile solvent for these target compounds, ethylene glycol being the best solvent. Among alkanediols, propanediols (1,2 and 1,3 isomers) stand out if both extraction yield and bioactivity studies are considered. Their extraction efficiency was close to ethanol, and their extracts also presented similar cytotoxicity, being active against the human cervical carcinoma cell line but not against the non-tumor porcine liver primary cell culture. None of the aqueous mixtures of solvents showed anti-inflammatory activity. Regarding the studied solvents (without extracts), those having up to four carbon atoms were not toxic for the selected cell cultures in the studied concentration range.

The results obtained suggest the use of the extracts prepared in 1,2 and 1,3-propanediol as a source of important phytochemicals with bioactive properties. Depending on the envisioned application, further tests should be carried out to ensure the safety of the solvents and extracts, while optimizing the extraction conditions.

## Figures and Tables

**Table 1:** Overview of the use of glycerol and alkanediols for the extraction of phenolic compounds from plant material.

Plant Material	Origin	Solvent	Technique	Main Compounds	Bioactivities	References
Glycerol-based solvents						
*Artemisia arborescens* L. *Artemisia inculta* Delile (aerial parts)	Greece	Glycerol + water (90%, *w*/*v*)	Stirring + heating	Total phenolic content Phenolic acids Flavonoids Others	Antioxidant *Ferric reducing power* *DPPH*	[27]
*Hypericum perforatum* L. (aerial parts)	Germany	Glycerol + water (10%, *w*/*v*)	Stirring + heating	Total phenolic content Phenolic acids Flavonols	Antioxidant *Ferric reducing power*	[26]
*Olea europaea* L. (leaves)	Greece	Glycerol + water (9.3%, *w*/*v*)	Stirring + heating	Total phenolic content Flavonoids Others	-	[28]
*Origanum onites* L. *Origanum vulgare* spp. hirtum *Origanum vulgare* L. (herbs)	Lithuania Turkey	Glycerol + ethanol (80–100%, *v*/*v*) Ethanol + water (30–96%, *v*/*v*) Methanol	UAE Heat-reflux Stirring Maceration Percolation	Rosmarinic acid Others	-	[16]
*Oryza sativa* L. (rice bran)	China	Glycerol + water (19.47%)	Shaking + heating	Total phenolic content Phenolic acids	-	[30]
*Solanum melongena* L. (peels)	Greece	Glycerol + water (90%, *w*/*v*) Ethanol + water (40%, *v*/*v*)	UAE	Total phenolic content Phenolic acids Flavonoids	Antioxidant *Ferric reducing power* *DPPH*	[29]
Propylene-glycol-based solvents						
*Calendula officinalis* L. (flowers)	Brazil	Propylene glycol + ethanol + water (40% + 40% + 20%, *v*/*v*/*v*)	Shaking	Phenolic acids Flavonoids	-	[17]
*Mellissa officinalis* L. *Origanum vulgaris* L. *Salvia officinalis* L. (herbs)	Slovakia	Ethanol + propylene glycol	Commercial extracts	Total phenolic content	Antioxidant *ABTS* *TBARS*	[21]
*Malus sylvestris* (L.) Mill. (wild fruit)	Serbia	Ethanol + water (70%, *v*/*v*) Propylene glycol + water (45%, *w*/*w*) Propylene glycol + water (8%, *w*/*w*) Water	Maceration Percolation Soxhlet Ultrasonic	Total phenolic content Total flavonoids Total tannins	Antioxidant *DPPH* *Ferric reducing power* *Inhibition of linoleic acid oxidation*	[18]
*Origanum onites* L. *Origanum vulgare* spp. hirtum *Origanum vulgare* L. (herbs)	Lithuania Turkey	Propylene glycol + ethanol (70–90%, *v*/*v*) Ethanol + water (30–96%, *v*/*v*) Methanol	UAE Heat-reflux Stirring Maceration Percolation	Rosmarinic acidOthers	-	[16]
*Pandanus amaryllifolius* Roxb. (leaf and root)	Thailand	Propylene glycol Ethanol (95%) Propylene glycol + ethanol (1:4 and 1:1, *v*/*v*)	Maceration	Total phenolic content	Antioxidant *DPPH* *Linoleic acid emulsion–thiocyanate method*	[20]
*Stevia rebaudiana* Bert. (leaf)	Poland	Propylene glycol + water (4:1) Ethanol (96%)	Stirring	Total phenolic content Phenolic acids Flavonoids	Antioxidant *DPPH* *ABTS* *Ferric reducing power* Cytotoxicity *CRL-2522*	[22]
*Terminalia chebula* Retz (dried fruits)	Thailand	Ethanol + water (76.4%, *v*/*v*) 1 Propylene glycol + water (36%, *v*/*v*)	Reflux	Total phenolic content Gallic acid Ellagic acid	Antioxidant *ABTS*	[24]
*Terminalia cheubula* Retz (fruits)	Thailand	Ethanol + water (30%, 50%, 70%, and 100%) Propylene glycol + water (30%, 50%, 70%, and 100%)	Maceration	Total phenolic content	Antioxidant *DPPH* *H2O2 inhibition* *AAPH-induced haemolysis* *ABTS* *Photochemiluminescence*	[23]
*Vitis vinifera* L. (pomace)	Italy	Propylene glycol + ethanol (1:1 and 1:3, *v*/*v*)	Stirring	Total phenolic content Gallic acid Flavonoids	Antioxidant *DPPH* *AAPH-induced haemolysis*	[19]
Ethylene glycol-based solvents						
*Morus alba* L. (leaf)	Korea	Ethylene glycol + water (25%, 42%, and 58%) Acetone + water (47% and 57%) Acetone + methanol (27%)	Heat extraction	Total phenolic content	Antioxidant *DPPH*	[31]

UAE: ultrasound-assisted extraction; DPPH: 2,2-diphenyl-1-picrylhydrazyl; ABTS: 2,2′-azino-bis-3-ethylbenzthiazoline-6-sulphonic acid; TBARS: thiobarbituric acid reactive substances; CRL-2522: human skin fibroblasts BJ; AAPH: 2,2′-Azobis(2-amidinopropane) dihydrochloride.

**Table 2:** Quantification of the main phenolic compounds present in different extracts of *J. regia* leaves (mean ± SD): 3-*O*-caffeoylquinic acid, *trans* 3-*p*-coumaroylquinic acid, quercetin 3-*O*-glucoside, quercetin *O*-pentoside, and total HPLC content.

Solvent	3-*O*-Caffeoylquinic Acid	*trans* 3-*p*-Coumaroylquinic Acid	Quercetin 3-*O*-glucoside	Quercetin *O*-pentoside	Total HPLC
	(mg/g Dry Plant)	(mg/g Dry Plant)	(mg/g Dry Plant)	(mg/g Dry Plant)	(mg/g Dry Plant)
water	5.16 ± 0.06b	1.07 ± 0.03d	4.3 ± 0.2h	3.59 ± 0.07h	14.1 ± 0.3g
ethanol	4.52 ± 0.01d	1.23 ± 0.02b	11.7 ± 0.1c	10.32 ± 0.06c	27.8 ± 0.1b
1,2-ethanediol	5.79 ± 0.08a	1.36 ± 0.02a	12.6 ± 0.1b	10.73 ± 0.08b	30.5 ± 0.2a
1,2-propanediol	4.96 ± 0.06c	1.14 ± 0.01c	11.3 ± 0.2d	9.8 ± 0.2d	27.2 ± 0.4c
1,3-propanediol	5.30 ± 0.04b	1.22 ± 0.04b	11.1 ± 0.1d	9.6 ± 0.07de	27.3 ± 0.3c
1,3-butanediol	4.46 ± 0.01d	1.03 ± 0.01d	9.85 ± 0.2f	8.6 ± 0.2f	23.9 ± 0.3e
1,2-pentanediol	1.30 ± 0.02g	0.300 ± 0.002g	13.0 ± 0.3a	11.45 ± 0.2a	26.0 ± 0.5d
1,5-pentanediol	4.47 ± 0.07d	1.22 ± 0.01b	10.75 ± 0.2e	9.5 ± 0.1e	25.9 ± 0.4d
1,2-hexanediol	3.00 ± 0.05f	0.8 ± 0.03f	1.93 ± 0.04i	nd	5.73 ± 0.09h
glycerol	4.3 ± 0.1e	0.94 ± 0.04e	6.98 ± 0.2g	6.1 ±0.1g	18.3 ± 0.4f

Calibration curves: 3-*O*-caffeoylquinic acid: 5-*O*-caffeoylquinic acid (y = 118879x–181046; *r*^2^ = 0.9992; limit of detection (LOD) = 0.20 µg/mL; limit of quantitation (LOQ) = 0.68 µg/mL); *trans* 3-*p*-coumaroylquinic acid: *p*-coumaric acid (y = 120011x+1×10^6^; *r*^2^ = 0.9973; LOD = 0.68 μg/mL; LOQ = 1.61 μg/mL); quercetin 3-*O*-glucoside and quercetin *O*-pentoside: quercetin 3-*O*-glucoside (y = 98385x + 143369; *r*^2^ = 0.9978; LOD = 0.21 μg/mL; LOQ = 0.71 μg/mL). nd.: not detected. Different letters represent significant differences (*p* < 0.05).

**Table 3:** Bioactive properties of *J. regia* leaves. Cytotoxicity in human tumor cell lines HeLa and non-tumor liver primary cells PLP2 (GI_50_). Quantification for extracts and solvents (mean ± SD).

Solvent	HeLa		PLP2	
	Extract (µg/mL)	Solvent (%, *v*/*v*)	Extract (µg/mL)	Solvent (%, *v*/*v*)
Water	>500	>4	>500	>4
Ethanol	245 ± 14b	>4	>500	>4
1,2-ethanediol	97 ± 10e	>4	142 ± 5b	>4
1,2-propanediol	292 ± 24a	>4	>500	>4
1,3-propanediol	216 ± 10c	>4	>500	>4
1,3-butanediol	257 ± 12b	>4	>500	>4
1,2-pentanediol	151 ± 12d	0.63 ± 0.04a	232 ± 13a	0.89 ± 0.04a
1,5-pentanediol	212 ± 4c	0.49 ± 0.02b	141 ± 3b	0.59 ± 0.02b
1,2-hexanediol	37 ± 2f	0.36 ± 0.02c	48 ± 5b	0.285 ± 0.005c
Glycerol	88 ± 4e	>4	143 ± 5b	>4

Positive controls: Ellipticine (GI_50_ values): HeLa: 1.03 ± 0.09 µg/mL; PLP2: 2.3 ± 0.2 µg/mL. Results expressed in mean values ± standard deviation (SD). Different letters represent significant differences (*p* < 0.05).
